# Quantitative Analysis of Retinal Vasculature in Rhegmatogenous Retinal Detachment Based on Ultra-Widefield Fundus Imaging

**DOI:** 10.3389/fmed.2021.797479

**Published:** 2022-01-18

**Authors:** Bingkai Feng, Wenxin Su, Qingshan Chen, Run Gan, Mingxuan Wang, Jiantao Wang, Jiayi Zhang, Xiaohe Yan

**Affiliations:** ^1^Shenzhen Key Laboratory of Ophthalmology, Shenzhen Eye Hospital, Shenzhen Eye Hospital Affiliated to Jinan University, Jinan University, Shenzhen, China; ^2^Institutes of Brain Science, State Key Laboratory of Medical Neurobiology, MOE Frontiers Center for Brain Science, Fudan University, Shanghai, China; ^3^Department of Psychology, University of Essex, Colchester, United Kingdom; ^4^Department of Biomedical Engineering, Johns Hopkins University, Baltimore, MD, United States

**Keywords:** retinal vasculature, rhegmatogenous retinal detachment, morphology analysis, quantitative analysis, confocal scanning laser ophthalmoscopy (cSLO)

## Abstract

**Purpose:**

To quantitatively analyze retinal vascular morphological features, such as vascular density, caliber, and tortuosity, in rhegmatogenous retinal detachment (RRD).

**Methods:**

A total of 244 patients with RRD and 400 healthy controls (HC) were included. Retinal fundus images were collected using OPTOS PLC Daytona P200T. Retinal images were divided into RRD and non-RRD regions of interest (ROIs). All visible retinal fundus vessels were then extracted mainly based on edge detection within ROI to form the whole-vascular image. Retinal vasculature parameters, such as vascular density, caliber, and tortuosity, were calculated.

**Results:**

For the absolute density, the mean rank (MR) value of normal controls was significantly higher than that in non-RRD (*p* < 0.001). A consistent tendency of significant vascular density was increased from non-RRD to RRD (*p* < 0.001). The average and median diameters of normal controls were both significantly larger than RRD (*p* < 0.001). The average and median diameters were also appeared significantly thinner in non-RRD. Unweighted and width-inversely-weighted vascular tortuosity in RRD and non-RRD comparison exhibited non-significant differences. All types of tortuosity calculated from HC were significantly larger (*p* < 0.001) in values compared to RRD. All types of tortuosity values of HC were significantly higher than non-RRD. Compared with non-RRD, RRD was significantly larger in area-weighted, length-weighted, and width-weighted vascular tortuosity.

**Conclusions:**

This study showed that RRD affects both the quantity and morphology of retinal vasculature, such as RRD and non-RRD areas. Smaller average and medium vascular diameters and tortuosity values were found in RRD. However, the absolute vascular density, the average and median diameter, and tortuosity values were also reduced in non-RRD although the retina is still attached. This work indicates that RRD may affect the retinal vasculature beyond the detached retina.

## Introduction

Retinal detachment (RD) is a vision-threatening medical emergency, in which the neurosensory retina is separated from the retinal pigment epithelium (RPE). It often presents with a central or peripheral painless vision loss, flashes, or floaters. Rhegmatogenous retinal detachment (RRD) is the most common category of RD caused by a subretinal fluid due to retinal break, with an annual incidence of 13.3–42 cases per 100,000 inhabitants (1–4). The diagnosis of RRD is based on fundus examination, by which the range of RD, retinal breaks, pigmented cells in the vitreous cavity, and pigmented demarcation line are found (5, 6). The ultra-widefield fundus (UWF) imaging system, Optos confocal scanning laser ophthalmoscopy (cSLO), can image up to 200° or 82% of the retina in one single capture, which is much greater extent than traditional fundus photography, helpful in screening or monitoring the progression of RRD during follow-up (7). Several fluorescein angiography (FA) studies of RRD confirmed the peripheral retinal vascular anomalies, such as blood flow reduction, vascular occlusions, arteriovenous shunts, and permeability alterations, which may be associated with lattice degeneration and retinal breaks (8–10). In addition, optical coherence tomography (OCT)-angiography (OCT-A) can provide non-invasive imaging of real-time retinal and choroidal vasculature blood flow, the vascular density of choroid superficial capillary plexus is lower in the eye with RRD involved in macula than the fellow eye and larger foveal avascular zone is related to worse visual acuity (11). These studies showed that retinal vascular morphology may be changed in RRD, such as the non-RRD area. But the details remain unknown, especially the retinal vessel features in the non-RRD area in RRD. The quantitative analysis of retinal vascular morphological features, such as vascular density, caliber, and tortuosity, can help to develop artificial intelligence screening and understand the pathogenesis of RRD. In this study, we evaluated retinal vasculature parameters in patients with RRD by a cascaded deep-learning system based on the UWF images.

## Methods

### Participants

This study was approved by the Medical Ethics Committee of Shenzhen Eye Hospital, Shenzhen, China. A total of 400 healthy control (HC) participants and 244 patients with pre-operative RRD were included. All retinal fundus images were collected using Optos cSLO (OPTOS PLC Daytona P200T) with a field of view of 200°.

### Sample Size Calculation

The highest incidence rate of RD was 0.042%, which WAS mentioned in the previous literature (1). We calculated the expected sample size by setting a confidence level as 95%, the margin of error as 5% using the formula below, $n=\;\frac{z^{2}\times p\;\left(1-p\right)}{\varepsilon^{2}}$

*n* represents the expected minimum sample size, *z* is a score transferred from a confidence level, *p* is the population proportion, and ε is the margin of error (12).

### Regions of Interest (ROIs) for Images

**Figure 1:**
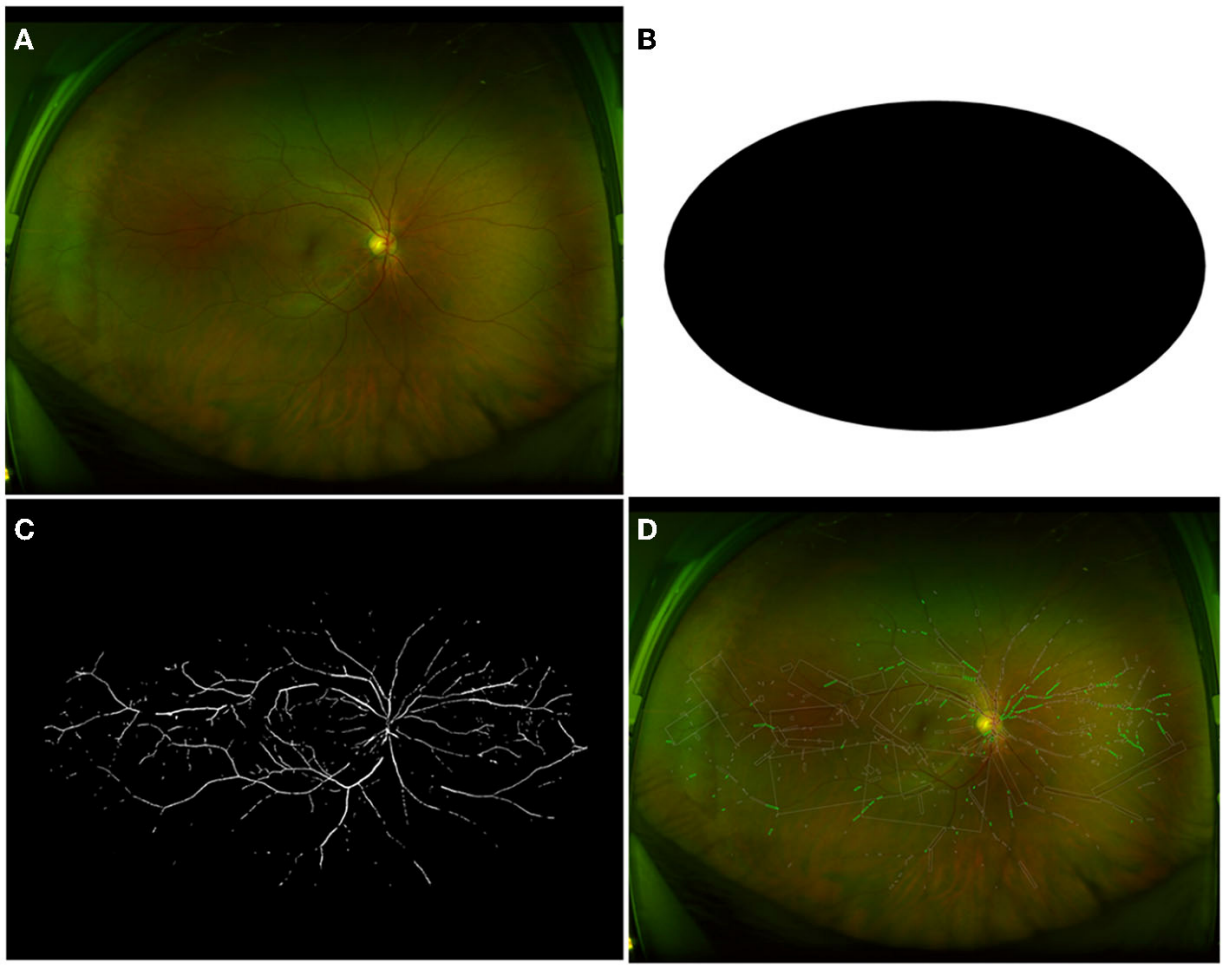
Image process of HC group with general cover. **(A)** The raw retinal image of one participant from the HC group. **(B)** Displayed general cover to get rid of inferences of eyelid and eyelash. **(C)** Extracted vessels as a binary image to compute vessel density. **(D)** The calculation for vessel caliber and tortuosity. HC, healthy controls.

**Figure 2:**
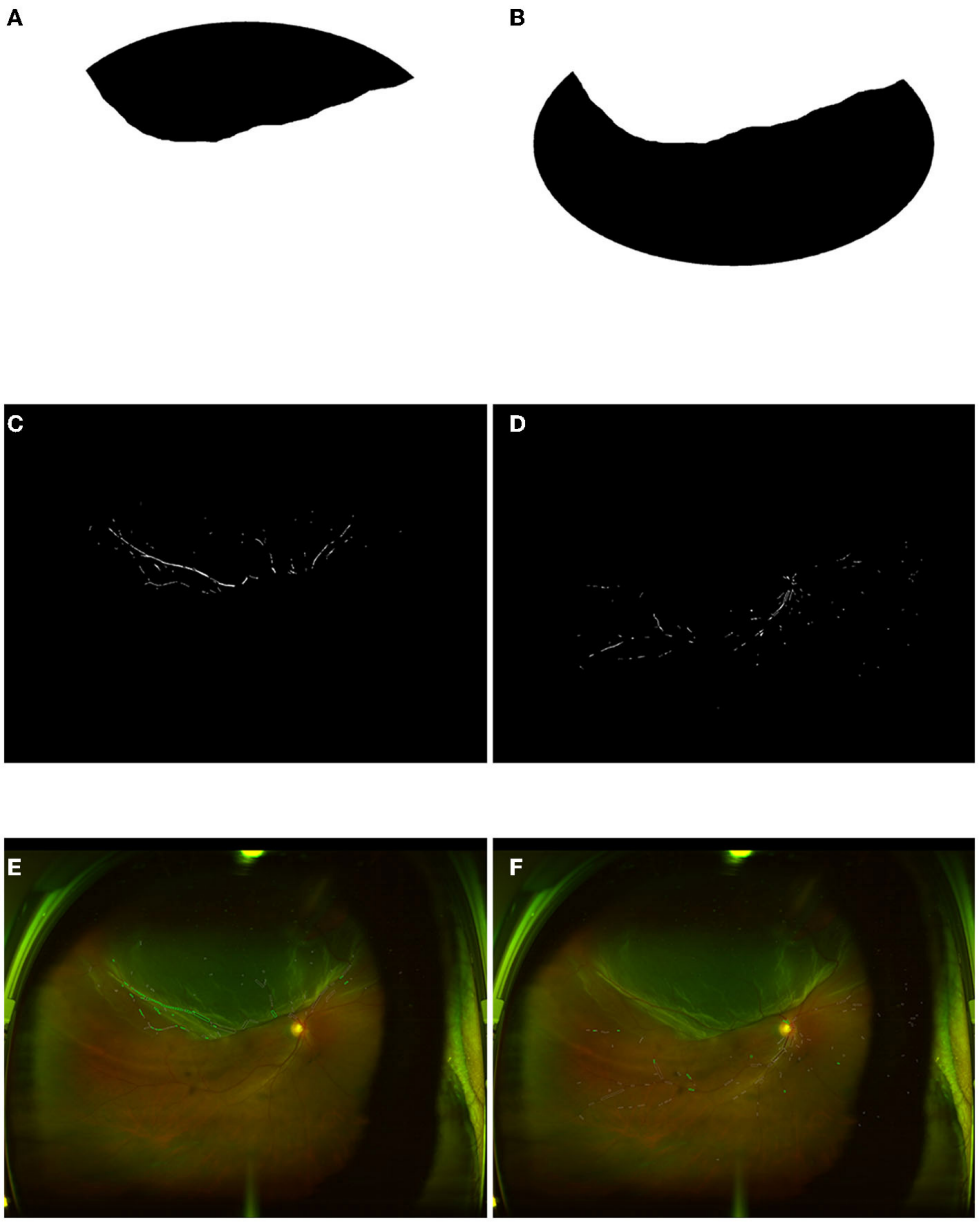
Image process of RRD and non-RRD group with personality cover. **(A)** Personality cover for RRD part from one retinal image. **(B)** Personality cover for a non-RRD part from the same retinal image. **(C,D)** Extracted vessels as a binary image to compute vessel density from RRD and non-RRD parts. **(E,F)** The calculation for vessel caliber and tortuosity from RRD and non-RRD parts. RRD, rhegmatogenous retinal detachment.

Due to the inference of eyelid and eyelash, we set up a general cover with an oval of about 43% proportion in the image at the geometric center of images. Therefore, ROIs for images from HC participants are selected by this general cover, as shown in Figure 1B. An example of the raw retinal image from the HC group, corresponding extracted vessels, and the visualized computational results for vasculature parameters were shown in Figures 1A,C, and D. For retinal detached patients, their image ROIs would be further selected by specialized covers, as shown in Figures 2A,B. Those covers were produced by doctors in Shenzhen Eye Hospital by drawing boundaries on each image which distinguish the RD region and the rest. Therefore, retinal images of the patients were divided into RRD ROIs and non-RRD ROIs for further analysis. The example of separate personalized classified vessels (Figure 2C for RRD-ROIs and Figure 2D for non-RRD-ROIs) and visualized vasculature parameters on related images (Figure 2E for RRD-ROIs and Figure 2F for non-RRD-ROIs) from one patient were shown in Figure 2.

### Digitization of Images

The digitization process aims at the automatic extraction of vessels from retinal fundus images. Using filters from two-color representation spaces, Hue, Saturation, and Value (HSV) and Red, Green, and Blue (RGB), we managed to extract different aspects of the character from the image. All images were pre-processed by HSV filter, which displayed superiority in sharpening bright area as represented by the optic disk when the value-channel (brightness) information is kept in the grayscale image. RGB filter enhanced the contrast between blood vessels and the background when the red-channel information was removed (red-free), whereas differences between the brightness of arteries and veins are most notable under green-channel (green-only). The red-free and green-only images were converted into the grayscale images, respectively. All visible retinal fundus vessels were then extracted mainly based on edge detection within ROI to form the whole-vascular image.

### Calculation of Vasculature Parameters

The statistical analysis of vessels was based on vasculature parameters. They are vascular density, caliber, and tortuosity. Detailed calculation method could be found in Wang et al. (13).

### Calculation of Vascular Density-Related Parameters

Absolute density was calculated based on the number of pixels within vessels, divided by corresponding ROIs. For the images of the patients, RRD and non-RRD ROIs were specialized settled for individual image. As for HC images, their ROI areas were the oval in the general cover.

### Calculation of Vascular Caliber-Related Parameters

Vascular caliber-related parameters were computed based on segmented vessels and qualification of vascular segments. Criteria about inner segment's area and enclosing rectangle area; length of the long side of segment's minimum enclosing rectangle; and acute angle between this and the line passing through optic disk center; and the distance between nearest neighboring circle and optic disk center were settled to select vessel segments.

The width of each caliber-computable vascular segment's minimum enclosing rectangle was considered the diameter of this vascular segment. Average and median diameters of all caliber-computable vascular segments were then computed as vascular average caliber and median caliber. These two parameters were computed on whole-vascular for all three groups of images, respectively, to acquire 2 vascular caliber-related parameters of each image.

### Calculation of Vascular Tortuosity-Related Parameters

The computation of vascular tortuosity-related parameters relied on the identification of tortuosity-computable vascular segments and corresponding adjacent vascular segments. Such criteria were also similar with the qualification of computable caliber segments.

Varies of tortuosity parameters were applied to capture different aspects of vascular tortuosity. They were unweighted, area-weighted, length-weighted, width-weighted, and width-inversely-weighted vascular tortuosity. Area-weighted tortuosity was a parameter combined length and width weighting information. Length-weighted tortuosity was mainly contributed as a calibration for bias due to small and large vessels due to the segmentation process. Width-weighted tortuosity was applied to eliminate different impacts on tortuosity due to the variant of vascular caliber. However, the width-inversely-weighted vascular tortuosity aimed at inversely eliminating the different impacts on tortuosity for the caliber variation compared to the width-weighted tortuosity. Equations for the computation process were mentioned below.

Unweighted vascular tortuosity *T*_*normal*_ was computed as follows:


$$\begin{array}{l} \{\begin{array}{c} \theta_{x}=\frac{\theta_{x}}{2}\;,\;\text{type}\;II\;segment\;\\ \theta_{y}=\frac{\theta_{y1}\;+\;\theta_{y2}}{2},\;\text{type}\;III\;segment\; \end{array}\\ \;T_{normal}=\frac{1}{n}•\sum_{i=1}^{i=\;n}\theta_{i}, \end{array}$$

θ_*x*_ represents the inter-segment angle of marginal vascular segment *x*, which is the acute angle between the lines parallel to the long sides of segment *x* and its unique adjacent segment's minimum enclosing rectangles, respectively. θ_*y*_ represents the inter-segment angle of intermediate vascular segment *y*, while θ_*y*1_ and θ_*y*2_ were acute angles between the line parallel to the long side of segment *y*'s minimum enclosing rectangle and the lines parallel to the long sides of its two adjacent segments *y*1 and *y*2's minimum enclosing rectangles, respectively. *n* was the number of marginal and intermediate vascular segments. θ_*i*_ was the inter-segment angle of vascular segment *i*.

Area-weighted vascular tortuosity *T*_*area*_ was computed as follows to compensate for the unevenness of divided vascular segments (see reference 12 for details).

Length-weighted vascular tortuosity *T*_*length*_ was computed as follows to adjust variance among lengths of vascular segments (see reference 12 for details).

Width-weighted vascular tortuosity *T*_*width*_ was computed as follows to amplify the tortuosity of thick vessels (see reference 12 for details).

Width-inversely-weighted vascular tortuosity *T*_*widt*_*h*__*inv*__ was computed as follows to mainly reveal thin vessels' tortuosity (see reference 12 for details).

### Statistical Analysis

Both comparisons of vessel parameters between HC vs. RRD ROIs, HC vs. non-RRD ROIs were performed by the non-parametric independent sample Mann-Whitney U test. On the contrary, comparisons of vessel parameters between RRD vs. non-RRD ROIs were applied by the non-parametric Wilcoxon signed-rank 2-tail test.

## Results

**Figure 3:**
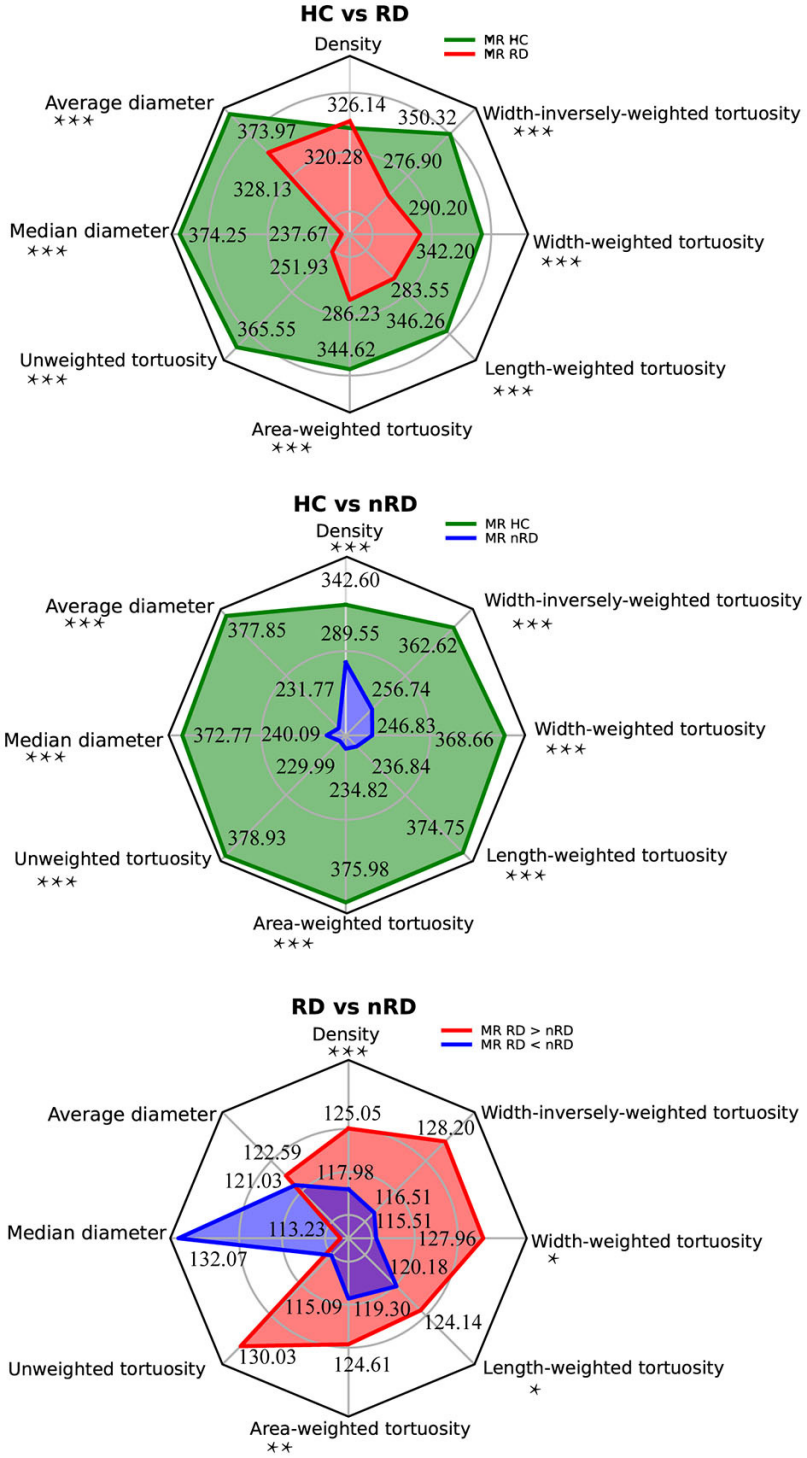
Group comparisons among HC, RRD, and non-RRD groups of all vascular parameters. The three radar plots displayed all vascular parameters from the top with anti-clockwise order, they are density, average diameter, and median diameter to evaluate vascular caliber; unweighted tortuosity, area-weighted tortuosity, length-weighted tortuosity, width-weighted tortuosity, and width-inversely-weighted tortuosity to measure vascular tortuosity. The top panel represented mean rank results from the comparison between HC and RRD groups by the Mann-Whitney test, HC in green and RRD in red. The middle panel showed mean rank results between HC and non-RRD groups by the Mann-Whitney test, HC in green and non-RRD in blue. The bottom panel illustrated results from the Wilcoxon signed-rank 2-tail test between RRD and non-RRD comparison, whereas ranks which RRD larger than non-RRD in red and ranks which RRD smaller than non-RRD in blue. In all three comparisons, *n*_HC_ = 400, *non-RRD* = 244, and *n*_non−RRD_ = 244. *p < 0.05, **p < 0.01, ***p < 0.001. HC, healthy controls; RRD, rhegmatogenous retinal detachment.

The computed sample size was almost equal to 1 due to the relatively small incidence rate. Therefore, the number of cases in the present study largely exceeded the needs. A summary of the statistical result was illustrated below as radar plots (Figure 3). Further details were shown later.

### Vascular Density Analysis

As measured by absolute density, the mean rank (MR) value of the HC in our study was 342.60, which was significantly higher than that in non-RRD (MR = 289.55) with Mann-Whitney *U* scored as 40761.00 (*n*_*HC*_ = 400, *n*_*non*−*RRD*_ = 244, *p* < 0.001). Meanwhile, we speculated a consistent tendency of significant vascular density increased from non-RRD (*MR*_*RD*<*non*−*RRD*_ = 117.98) to RRD (*MR*_*RD*>*non*−*RRD*_ = 125.05) by the Wilcoxon test (*n* =244, *Z* = −4.14*, p* < 0.001).

In summary, non-RRD ranks the smallest in the variation of absolute density among all HC, non-RRD, and RRD.

### Vascular Caliber Analysis

Average and median diameters of HC (*MR*_*average*_ = 373.97, *MR*_*median* =_ 374.25) were both significantly larger than RRD (*MR*_*average*_ = 238.13, *MR*_*median* =_ 237.67) with the Mann-Whitney test (*U*_*average*_ = 28212.50, *U*_*median* =_ 28101.50, *n*_*HC*_ = 400, *non-RRD* = 244, *p* < 0.001). Unsurprisingly, both caliber measurements, average and median diameters reflected common tendency. Moreover, the average and median diameters were also appeared significantly thinner (*U*_*average*_ = 26662.00, *U*_*median* =_ 28693.00, *n*_*HC*_ = 400, *n*_*non*−*RRD*_ = 244, *p* < 0.001) in non-RRD (*MR*_*average*_ = 231.77, *MR*_*median* =_ 240.09) compared with HC (*MR*_*average*_ = 377.85, *MR*_*median*_ = 372.77).

However, no significant difference was found between RRD and non-RRD in vascular caliber-related parameters by the Wilcoxon non-parametric test, only a slightly tendency of increase from non-RRD (*MR*_*RD*<*non*−*RRD*(*average*)_ = 121.03, *MR*_*RD*<*non*−*RRD*(*median*)_ = 132.07) to RRD (*MR*_*RD*>*non*−*RRD*(*average*)_ = 122.59, *MR*_*RD*>*non*−*RRD*(*median*)_ = 113.23) was observed in both average and median diameter measures.

Taking together, our findings only suggest that HC led the highest value of vascular caliber among all three groups.

### Vascular Tortuosity Analysis

Unweighted and width-inversely-weighted vascular tortuosity in RRD and non-RRD comparison exhibited non-significant differences. All types of tortuosity calculated from HC (*MR*_*unweighted*_ = 365.55; *MR*__*area*_−weighted_ = 344.62; *MR*_*length*−*weighted*_ = 346.26; *MR*_*width*−*weighted*_ = 342.20; *MR*_*width*−*inversely*−*weighted*_ = 350.32) were always discovered to be significantly larger (*U*_*unweighted*_ = 31580.50; *U*_*area*−*weighted*_ = 39950.50; *U*_*length*−*weighted*_ = 39296.50; *U*_*width*−*weighted*_ = 40919.50; *U*_*width*−*inversely*−*weighted*_ = 37674.00, *n*_*HC*_ = 400, *non-RRD* = 244, *p* < 0.001) in values compared to RRD (*MR*_*unweighted*_ = 251.93; *MR*_*area*−*weighted*_ = 286.23; *MR*_*length*−*weighted*_ = 283.55; *MR*_*width*−*weighted*_ = 290.20; *MR*_*width*−*inversely*−*weighted*_ = 276.90), no matter which weighting method was conducted.

Similar to comparisons mentioned above, we found all types of tortuosity values of HC (*MR*_*unweighted*_ = 378.93; *MR*_*area*−*weighted*_ = 375.98; *MR*_*length*−*weighted*_ = 374.75; *MR*_*width*−*weighted*_ = 368.66; *MR*_*width*−*inversely*−*weighted*_ = 362.62) significantly higher (*U*_*unweighted*_ = 26228.50; *U*_*area*−*weighted*_ = 27406.50; *U*_*length*−*weighted*_ = 27899.50; *U*_*width*−*weighted*_ = 30337.50; *U*_*width*−*inversely*−*weighted*_ = 32754.50, *n*_*HC*_ = 400, *n*_*non*−*RRD*_ = 244, *p* < 0.001) than non-RRD (*MR*_*unweighted*_ = 229.99; *MR*_*area*−*weighted*_ = 234.82; *MR*_*length*−*weighted*_ = 236.84; *MR*_*width*−*weighted*_ = 246.83; *MR*_*width*−*inversely*−*weighted*_ = 256.74), regardless of weighting methods.

Compared with non-RRD, RRD was significantly larger in area-weighted, length-weighted, and width-weighted vascular tortuosity (Z_area−weighted_ = −3.06, *p*_*area*−*weighted*_ = 0.002; *Z*_*length*−*weighted*_ = −2.34*, p*_*length*−*weighted*_ = 0.019; *Z*_*width*−*weighted*_ = −2.54, *p*_*width*−*weighted*_ = 0.011; *n* = 244). In area-weighted tortuosity, *MR*_*RD*>*non*−*RRD*_ = 124.61, *MR*_*RD*<*non*−*RRD*_ = 119.30; whereas in length-weighted tortuosity, *MR*_*RD*>*non*−*RRD*_ = 124.14, *MR*_*RD*<*non*−*RRD*_ = 120.18. Last, in width-weighted vascular tortuosity, *MR*_*RD*>*non*−*RRD*_ = 127.96, *MR*_*RD*<*non*−*RRD*_ = 115.51.

In conclusion, the vascular tortuosity in HC was the largest among HC, RDD, and non-RDD. In addition, the area-weighted, length-weighted, and width-weighted vascular tortuosity suggested non-RRD group represents the lowest values in such measurement among all three groups.

## Discussion

This is the first study to quantitatively analyze retinal vascular morphological features, such as vascular density, caliber, and tortuosity, in RRD. Firstly, reduced average and medium vascular diameter and smaller tortuosity values were found in RRD compared to normal controls. Secondly, the absolute vascular density and some tortuosity values were significantly larger in RRD than non-RRD. Thirdly, the absolute vascular density, the average and median diameters, and tortuosity value were significantly smaller in non-RRD than normal controls.

Vessels are straight or slightly curved, with an appropriate ramification structure transporting blood with high efficiency and low energy (14). Retinal vasculature manifests as branching patterns, which sprout from the optic disk toward the periphery (15, 16). Alterations in the vascular network affect retinal blood flow, which may increase or decrease the risk of ischemia, playing a significant role in the pathogenesis of ocular diseases (17). There are several studies indicating that retinal vascular abnormalities may occur in RRD. A reduction of capillary density with pre-operative RRD was detected compared to the fellow unaffected eyes (11, 18). The circulation time was slower in the detached areas compared to the non-detached areas by video fluorescence angiography (19). Doppler sonography found worse flow parameters of the central retinal artery in pre-operative RRD eyes, influenced by detachment duration (20). In addition, blood flow was reduced in the optic nerve head in patients with RRD (21). These studies indicated that capillary density, morphology, and blood flow were affected in RRD. Except for retinal capillary, we found that the average and medium vascular diameters and tortuosity values of whole retinal vascular density in RRD were also reduced. Our results support that the risk of ischemia may be increased in the whole retina in RRD.

In addition, the absolute vascular density, the average and median diameters, and tortuosity values were significantly reduced in non-RRD compared to normal controls. Several studies showed that inflammatory cytokines were significantly increased in eyes with RRD (22–24). Endothelin-1 (ET-1), a vasoconstrictive peptide causing a vasoconstriction on the retinal microvasculature and a consequent reduction in blood flow, had a high level in subretinal fluid, and plasma after detachment and its receptors were additionally strongly expressed in retinal blood vessels (25–28). Besides, inflammation could active Muller cells in both detached and non-detached retina, which was found to be related with local blood flow alterations in the inner retina and could lead to a secondary reduction in capillary density (29, 30). Therefore, these factors could cause vasoconstriction on the retinal vessel, leading to a reduced vascular density and diameter, such as the vessel in the non-RRD area. Tortuosity is a key parameter indicating the vasculature optimality state and the level of ocular perfusion. In hypoxia conditions, the mediators secreted by vascular endothelial cells may be associated with autoregulating blood flow, increasing vascular tortuosity, and promoting better tissue perfusion (31, 32). The reduced vascular tortuosity in non-RRD may reflect a compensatory mechanism to increase blood flow into the RRD area or its vascular dynamic alteration caused by RRD.

The absolute vascular density and some tortuosity values were significantly larger in RRD than non-RRD. Similarly, earlier studies showed dilatation and hyperpermeability of capillaries in RRD by FA (9, 33). When separated from the RPE, the outer retina becomes ischemic due to the lack of blood supply from the choroid, which may lead to capillary dilatation (34). Sufficient blood flow and capillary network are important for maintaining the normal metabolic activities and integrity of the retina (35). Vascular biomechanical instability due to RD may also result in vascular functional and anatomical changes.

There were several limitations in this study. For UWF images, stereographic projection correction holds an important effect in vascular parameters measurement, which previous research studies observed the difference may be up to 14.8% (36). This problem will be exacerbated in the RD, since the detached retina brings irregular stereographic projection. In addition, a wider field means a lower sensitivity for visualizing smaller capillaries. When the capillary segments with a diameter of 6.1–7.0 μm were all visualized on FA, only 43% of the capillary segments with a diameter of 4.1–4.5 μm could be detected (37). The missing detection of micro-small vessels and capillaries may not well-enough to reflect the vascular injury of RRD. In addition, all participants were Chinese, the vascular status in patients of RRD in other races may differ.

## Conclusion

This study showed that RRD affects both the quantity and morphology of retinal vasculature, including RRD and non-RRD areas. Smaller, average, and medium vascular diameters and tortuosity values were found in RRD. However, the absolute vascular density, the average and median diameter, and tortuosity values were also reduced in non-RRD although the retina is still attached. This work indicates that RRD may affect the retinal vasculature beyond the detached retina.

## Data Availability Statement

The raw data supporting the conclusions of this article will be made available by the authors, without undue reservation.

## Ethics Statement

The studies involving human participants were reviewed and approved by Shenzhen Eye Hospital. Written informed consent for participation was not required for this study in accordance with the national legislation and the institutional requirements. Written informed consent was not obtained from the individual(s) for the publication of any potentially identifiable images or data included in this article. No potentially identifiable human images or data is presented in this study.

## Author Contributions

XY, JZ, and JW conceived and designed the experiments. BF and WS performed the experiments. BF, WS, JW, XY, and JZ analyzed the data. BF, WS, XY, and JZ wrote the manuscript. All authors contributed to the article and approved the submitted version.

## Funding

This study was supported by the grants JCYJ20200109145001814 from the Research Foundation of Science and Technology Plan Project, Shenzhen, China, KQJSCX20180329171913184 from the High-level Overseas Talents Research Project, Shenzhen, China, Shenzhen Fund for Guangdong Provincial High-level Clinical Key Specialties (No.SZGSP014), and 81970790, 31771195, 81790640, 82021002 from the National Natural Science Foundation of China, a Shanghai Municipal Science and Technology Major Project (2018SHZDZX01), ZJLab and Shanghai Center for Brain Science and Brain-Inspired Technology, Sanming Project of Medicine in Shenzhen (SZSM202011015), Research and Development Fund of Zhongshan Hospital (2020ZSFZ19).

## Conflict of Interest

The authors declare that the research was conducted in the absence of any commercial or financial relationships that could be construed as a potential conflict of interest.

## Publisher's Note

All claims expressed in this article are solely those of the authors and do not necessarily represent those of their affiliated organizations, or those of the publisher, the editors and the reviewers. Any product that may be evaluated in this article, or claim that may be made by its manufacturer, is not guaranteed or endorsed by the publisher.
